# Clinical and radiological outcomes of arthroscopically assisted cannulated screw fixation for tibial eminence fracture in children and adolescents

**DOI:** 10.1186/s12891-018-1960-7

**Published:** 2018-02-06

**Authors:** Chang Ho Shin, Doo Jae Lee, In Ho Choi, Tae-Joon Cho, Won Joon Yoo

**Affiliations:** Department of Orthopaedic Surgery, Seoul National University College of Medicine, 101 Daehak-ro Jongno-gu, Seoul, 03080 Republic of Korea

**Keywords:** Tibial eminence fracture, Cannulated screw, Arthroscopically assisted reduction, Skeletally immature patient

## Abstract

**Background:**

The purpose of this study was to determine the efficacy and complications of arthroscopically assisted reduction and fixation with cannulated screws for tibial eminence fracture in skeletally immature patients.

**Methods:**

This was a retrospective case series study. Forty-eight patients who were skeletally immature at the time of tibial eminence fracture were treated in a tertiary children’s hospital between May 2004 and August 2015. Twenty-one patients were excluded due to non-operative treatment (*n* = 10), other surgical treatments (*n* = 9), multiple fracture (n = 1), and follow-up < 1 year (n = 1). Twenty-seven knees of 27 patients were analyzed. Avulsed fragment was reduced arthroscopically. One to three cannulated screws (4.0 mm or 5.0 mm in diameter) were used for fixation. Passive knee motion was started in 3–4 weeks. Clinical outcomes were evaluated by Lysholm score, instability of the knee, and complications. Radiological outcomes including nonunion and malunion of the avulsed fragment and physeal growth disturbance were evaluated.

**Results:**

Mean age at the time of surgery was 10.1 years (range, 6.2 to 13.8 years). Patients were followed up for a mean of 3.9 years (range, 1.0 to 7.6 years). Fracture types included type III (*n* = 13), type II (*n* = 12), and type IV (*n* = 2) according to Zaricznyj modification of Meyers and McKeever classification. Meniscus was entrapped in five patients. Six patients showed concomitant meniscal tear. Mean Lysholm score at the latest follow-up was 95 (range, 78 to 100). Joint instability was not observed in any patient except one (instability of 5–10 mm). All patients showed full range of knee motion except one (10 degrees of flexion contracture). Screw head impingement against intercondylar notch of the femur was observed in two patients during screw removal procedure. Five knees showed prominent tibial eminence without symptoms. The injured lower limb was longer than the contralateral normal side by a mean of 6.2 mm (range, − 4 to 18 mm).

**Conclusions:**

Arthroscopically assisted reduction and fixation with cannulated screws is an effective and safe surgical option for treating tibial eminence fracture with few complications.

## Background

Tibial eminence fracture is a relatively rare injury, with an incidence of approximately 3 per 100,000 per year, which mainly occurs in children and adolescents [1]. It is thought to be a pediatric equivalent of anterior cruciate ligament (ACL) tears in adults [1–3]. Meyers and Mckeever classified this fracture into three types initially in 1959 while Zaricznyj added type IV fracture in 1977 [2, 4]. Non-displaced type I fracture and slightly displaced type II fracture can be managed conservatively with successful closed reduction, while displaced fracture requires surgical treatment [5, 6].

Various surgical fixation methods are available. Cortical screw, headless screw, suture using absorbable or non-absorbable material, suture anchor, or Kirschner wire can be used to fix the fracture [7–12]. As joint stiffness associated with delayed knee mobilization after surgery is the main complication in treatment of tibial eminence fracture [13–16], the ideal fixation device should provide sufficient initial fixation strength to allow a more aggressive rehabilitation protocol. In addition, the fixation device should not traverse the proximal tibial growth plate in skeletally immature patients because of the possibility of partial physeal arrest [17, 18]. Some biomechanical studies have investigated fixation power of several internal fixation devices with different results [19–24]. Some clinical studies have reported outcomes of various fixation devices and surgical techniques with pros and cons [9, 10, 25–29]. Cannulated screw fixation has been used widely in the treatment of the tibial eminence fracture [27, 30–34]. However, only a few articles have studied this subject in skeletally immature patients who are different from adult patients in terms of knee size, screw length, and number of screws implanted [27, 33, 34].

The purpose of this study was to determine the efficacy and complications of arthroscopically assisted reduction and fixation with cannulated screws for tibial eminence fracture in skeletally immature patients.

## Methods

This retrospective study was approved by our Institutional Review Board (H-1611-007-803). A total of 48 patients who sustained tibial eminence fracture before skeletal maturity and were treated in a tertiary children’s hospital between May 2004 and August 2015 were reviewed. Twenty-one patients were excluded because of non-operative treatment (*n* = 10), other surgical treatments (*n* = 9, arthroscopic suture fixation in six patients, open reduction and fixation with cannulated screws in one patient, arthroscopic partial excision of tibial eminence and femoral intercondylar notchplasty in one patient, and arthroscopic exploration in one patient), multiple fractures in other body parts (*n* = 1), and follow-up of less than 1 year (n = 1). After excluding these patients, 27 knees of 27 patients remained eligible for inclusion.

### Surgical technique

All operations were performed by a single surgeon (W.J.Y.). In a fresh case of fracture, the hematoma was removed first and the avulsed fragment was pushed using a guide pin to achieve reduction while extracting the interposed meniscus or intermeniscal ligament using an arthroscopic probe. And then other guide pins were inserted before inserting a screw to prevent posterior displacement or rotation of the avulsed fragment. Guide pins were driven at a 30–50 degree sagittal plane angle to the growth plate of the proximal tibia. One to three ASNIS III cannulated screws (4.0 or 5.0 mm diameter; Stryker Corporation, Kalamazoo, MI, USA) were used with or without washers. Diameter and length of screws, number of screws implanted, and usage of washers were determined intraoperatively considering fracture pattern and the size of the avulsed osteochondral fragment and the knee. Intraoperative fluoroscopy was used to avoid iatrogenic physeal penetration by screws. In a delayed case of fracture, an arthroscopic burr and curette were used to remove the organized hematoma and fibrocartilaginous tissues on the old fracture surfaces before reduction and fixation of the avulsed fragment. In some delayed cases in which reduction was difficult to achieve, a temporary suture that had been passed through ACL was pulled out under meniscus to reduce the fragment, and a guide pin was inserted.

### Post-operative management/rehabilitation protocol

Postoperatively, a long leg splint was applied for 3–4 weeks with the knee in 30 degrees of flexion. Passive knee motion was then started. Weight bearing was permitted at 6 weeks after the surgery. Patients were allowed to return to sports at 3 to 4 months after the surgery.

### Clinical evaluations

Clinical outcomes were evaluated with regard to the presence or absence of arthrofibrosis, instability of the knee, Lysholm knee score [35], and complications including screw head impingement at the latest follow-up. Arthrofibrosis was defined as lack of 10 degrees or more of extension, or 15 degrees or more of flexion compared to the uninjured side at 3 months after treatment [13, 14]. Instability was evaluated using the side-to-side difference of Lachman and anterior drawer tests, and anterior tibial translation at 30 degrees of flexion in a KT-1000 arthrometer (MEDmetric, San Diego, CA, USA). Instability tests were performed by a single investigator (W.J.Y.). Lysholm knee score was obtained for 22 (81%) patients. KT-1000 measurements were obtained for 17 (63%) patients.

### Radiological evaluations

Radiological outcomes were evaluated with regard to penetration of proximal tibial growth plate by screws, nonunion and malunion of the avulsed fragment, and physeal growth disturbances such as leg length discrepancy (LLD) and angular deformity. LLD was assessed in 20 (74%) patients using standing whole leg anteroposterior (AP) radiograph at the latest follow-up. A patient who had suffered a femoral shaft fracture in the ipsilateral leg 5 years before tibial eminence fracture was excluded from the evaluation of LLD. Femoral length was determined by measuring the distance from the highest point of femoral head to the lowest point of medial femoral condyle. Tibial length was determined by measuring the distance from the medial tibial plateau to the tibial plafond on whole leg AP radiograph. Magnetic resonance imaging (MRI) study was taken preoperatively for all patients except two who were under Medical Aid Program. Based on MRI and arthroscopic findings, associated injury of the knee was evaluated.

### Statistical analysis

Mann-Whitney U test was used to compare means between two groups. To assess the relationship between the degree of laxity measured by KT-1000 or Lachman test and the Lysholm score, Spearman’s rank correlation test was used. *P* values of less than 0.05 were considered statistically significant.

## Results

Descriptive data of the 27 patients are summarized in Table 1. There were 19 boys and 8 girls. Their age at the time of surgery (mean ± standard deviation) was 10.1 ± 2.2 years (range, 6.2 to 13.8 years). Follow-up period was 3.9 ± 2.2 years (range, 1.0 to 7.6 years). The interval from trauma to surgery was 48.9 ± 143.0 days (range, 0 to 731 days). The interval was more than 30 days in 6 (22%) patients. Fractures were caused by fall (*n* = 11, 41%), sports injuries (*n* = 10, 37%, skiing in 8 patients, sprinting in 1 patient, and soccer in 1 patient), traffic accident (*n* = 2, 7%), or collision with another person (n = 2, 7%). Fracture type was type III in 13 (48%) patients, type II in 12 (44%) patients, and type IV in 2 (7%) patients according to Zaricznyj modification of Meyers and McKeever classification [4, 36].

**Table 1:** Descriptive Data and Results of 27 Patients

Patient	Age at surgery (years)	Age at the latest follow-up (years)	Interval from trauma to surgery (days)	Gender	Side	Type^a^	Cause of Injury	Anterior drawer/Lachman S-to-S diff (mm)	KT-1000 S-to-S diff (mm)	FC/FF at the latest follow-up (°)	Impingement	Lysholm score	Latest X-ray	LLD ICH^b^ (mm)	LLD FHH^b^ (mm)
1	11–12	18–19	12	F	Rt.	III	Fall	1/0		0/135	–	100	–		
2	3–4	14–15	7	F	Lt.	II	Fall	2/0		0/135	–	100	–		
3	8–9	16–17	141	M	Rt.	IV	RTA	0/0	4	−5/145	+	80	Ossicle	11	4
4	9–10	10–11	4	F	Lt.	III	Fall	0/0		−5/135	–		–		
5	8–9	13–14	4	F	Lt.	III	Collision with another person	0/0		0/135	–	100	–		
6	7–8	10–11	8	M	Lt.	II	Fall	0/0	1	−15/135	–	100	–	3	3
7	7–8	11–12	32	M	Lt.	III	Fall	0/0	1	10/130	+	85	Ossicle	−2	2
8	6–7	8–9	11	M	Lt.	II	Fall	0/1	2	−10/145	–	95	–	7	8
9	9–10	16–17	6	M	Lt.	II	Skiing	1/2		0/145	–	100	–	17	16
10	9–10	12–13	9	M	Rt.	III	Collision with another person	5/3	0	−5/150	–	100	–	13	7
11	6–7	11–12	731	M	Rt.	III	Running	3/0	0	0/140	–	93	Ledging	4	2
12	9–10	10–11	36	M	Rt.	II	Bicycling	5/9	5	0/140	–	78	–	10	7
13	10–11	15–16	3	F	Lt.	II	Skiing	3/0	1	−5/145	–	97	–	18	8
14	8–9	12–13	75	M	Rt.	IV	Fall	2/2		0/145	–	100	Ledging	11	9
15	10–11	11–12	5	M	Rt.	II	Fall	1/2	2	0/140	–	100	–	11	10
16	12–13	13–14	3	M	Rt.	II	Skiing	0/0		0/135	–		–		
17	11–12	18–19	0	M	Rt.	II	Fall	2/2	1	−10/135	–	91	Ledging	3	1
18	9–10	14–15	182	M	Rt.	III	Soccer	0/0	2	−5/135	–	100	Ledging	−4	−3
19	8–9	11–12	5	F	Lt.	III	Skiing	0/0		5/135	–		–	0	0
20	9–10	15–16	22	M	Rt.	III	Skiing	1/4	3	−5/135	–	95	–	8	8
21	12–13	16–17	1	F	Lt.	II	Skiing	0/0	2	0/135	–		–	−1	− 3
22	9–10	13–14	3	M	Rt.	II	Bicycling	3/3	1	−10/150	–	100	–	5	4
23	13–14	14–15	4	M	Rt.	III	Fall	0/0	2	−5/135	–	84	–	7	5
24	13–14	17–18	3	M	Rt.	III	Fall	2/0	2	−5/150	–	94	–		
25	13–14	15–16	2	M	Rt.	II	RTA	0/0		0/135	–	99	–	3.5	4.5
26	8–9	15–16	5	F	Lt.	III	Skiing	0/0		5/135	–		–		
27	10–11	14–15	5	M	Rt.	III	Skiing	0/2	2	−10/135	–	94	Ledging	0	−4

*S-to-S diff* Side-to-side difference; *FC* Flexion contracture; *FF* Further flexion; *LLD* Leg length discrepancy; *ICH* Iliac crest height; FHH, femoral head height; *RTA* Road traffic accident

^a^Zaricznyj modification of Meyers and McKeever classification, ^b^Positive value means that an affected leg is longer than a contralateral leg; Negative value means that a contralateral leg is longer than an affected leg

**Fig. 1:**
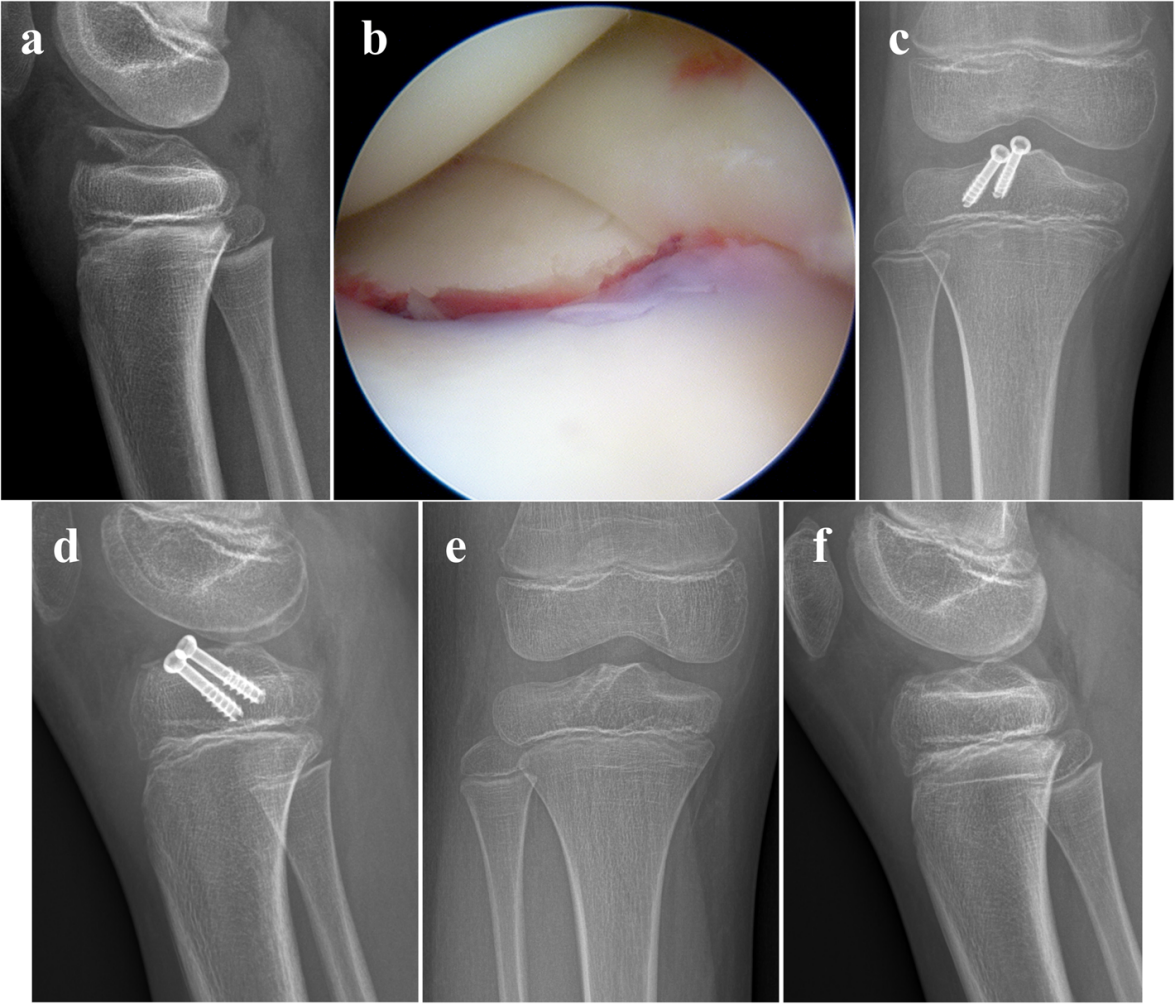
**a** A radiograph of a 10–11 year-old girl with tibial eminence fracture. **b** Anterior horn of medial meniscus entrapped under the avulsed fragment. **c, d** Radiographs taken at postoperative one month. The avulsed fragment was fixed with two 5.0 mm cannulated screws. **e, f** Radiographs taken at postoperative one year. Screws were removed. Her Lysholm knee screw was 100. She did not have instability of the knee

The number of screws used for fixation was one in three (11%) patients, two in 19 (70%) patients, and three in 5 (19%) patients. Washer was used in conjunction with the screw in 11 (41%) patients who had cartilaginous avulsion fragment with or without very thin bone sliver. The mean sagittal angle of the screw to the growth plate of the proximal tibia was 37.6 ° ± 8.0° (range, 20° to 53.4°). Five (19%) patients showed entrapment of the meniscus or intermeniscal ligament under a displaced tibial eminence fragment (intermeniscal ligament in 2 patients, medial meniscus in 2 patients, and lateral meniscus in 1 patient) (Fig. 1). Screw removal was routinely performed for all patients at postoperative 8.6 ± 5.9 months (range, 3.0 to 33.7 months).

### Clinical outcomes

**Fig. 2:**
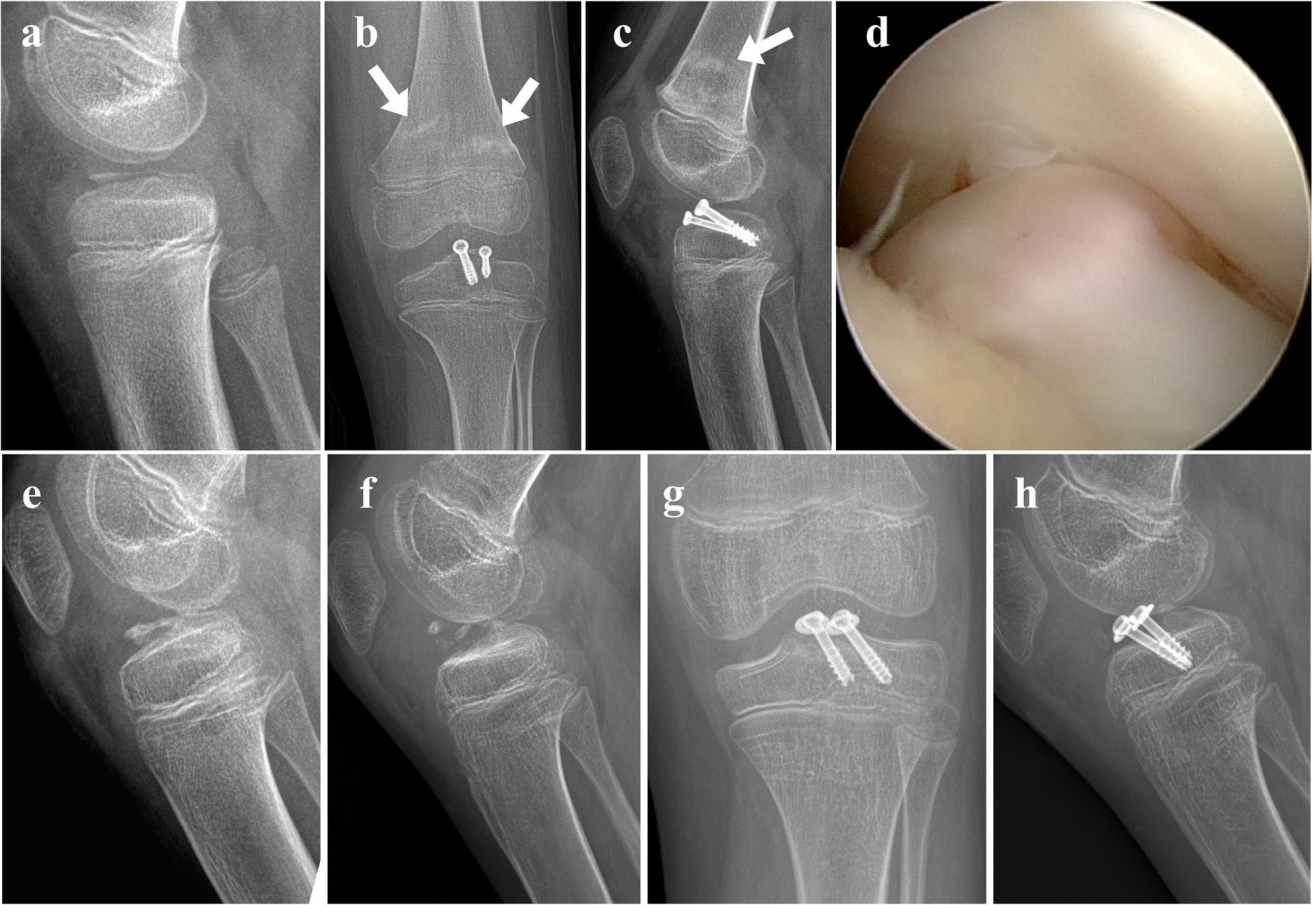
**a** A 8–9 year-old boy visited the clinic at 32 days post trauma with type III tibial eminence fracture. **b, c** Radiographs taken at postoperative 2.5 months. Incomplete distal femoral metaphyseal fracture (arrow) occurred during vigorous physical therapy. **d** While removing screws, it was found that the head of 5.0 mm screw impinged against the lateral portion of the medial femoral condyle and caused extension deficit of the knee. **e** Radiolucent gap between avulsed fragment and host bone was found on the day of screw removal. **f** A radiograph taken at postoperative 18 months. He was lost to follow-up for 1 year. He returned to the clinic with flexion contracture of 25° and re-displaced avulsed fragment. **g, h** He underwent open reduction and internal fixation with two 5.0 mm screws in conjunction with two washers for re-displaced fragments

Screw head impingement against femoral notch was observed in two (7%) patients during screw removal. Of these two patients, one patient was treated with one 4.0 mm screw and one 5.0 mm screw for neglected type IV fracture at 141 days after trauma. Hypertrophy of distal femoral articular cartilage was observed during screw removal procedure. This was probably due to irritation by screws. Range of knee motion was 15° to 120° before removal of screws and − 5° to 145° at the latest follow-up. The other patient was treated with one 4.0 mm screw and one 5.0 mm screw for type III fracture at 32 days post trauma (Fig. 2). Incomplete distal femoral metaphyseal fracture occurred due to vigorous physical therapy by mother at postoperative 2.5 months. This interrupted physical therapy for 3 weeks. Range of knee motion was 20° to 115° at postoperative 6 months when screws were removed. The head of 5.0 mm screw impinged against the lateral portion of medial femoral condyle and caused extension deficit of the knee. Range of motion of 10° to 120° could be easily obtained after screw removal. On post-removal radiograph, radiolucent gap between avulsed fragment and host bone was observed. This was thought to be a fibrocartilaginous union to lead to bony union which was expected. However, this patient was lost to follow-up at 3 months after screw removal. When he returned to the clinic at post-removal 18 months, he showed a 25° of flexion contracture. Plain radiographs revealed re-displaced avulsion fragment. Open reduction and internal fixation with two 5.0 mm cannulated screws with two washers were performed. At the latest follow-up, he showed a 10° of flexion contracture with further flexion of 130°. Bony union of avulsed fragment was achieved. There were no signs of knee instability. This patient was the only one who showed arthrofibrosis. All other 26 patients had full range of knee motion at the latest follow-up.

Side-to-side difference in Lachman test was less than 3 mm in 23 (85%) patients, 3 to 5 mm in 2 (7%) patients, and 5 to 10 mm in 1 (4%) patient. Side-to-side difference in anterior drawer test was less than 3 mm in 24 (89%) patients, 3 to 5 mm in 2 (7%) patients, and 5 to 10 mm in 1 (4%) patient. Of 17 patients who were examined with KT-1000 arthrometer, manual maximum side-to-side difference was less than 3 mm in 14 (82%) patients, 3 to 5 mm in 2 (12%) patients, and 5 mm in 1 (6%) patient. The patient who had 5 to 10 mm of side-to-side difference in physical examinations was the patient who showed 5 mm of side-to-side difference in the KT-1000 arthrometer measurement. He had been treated with one 4.0 mm screw and two 5.0 mm screws in conjunction with three washers for type II fracture at 36 days after trauma. Continuity of his ACL was maintained on preoperative MRI and arthroscopy. At the day of screw removal, however, the diameter of ACL decreased up to 50% of normal diameter, although the tension of the ligament was acceptable. He complained intermittent pain and swelling of the knee after sports activity, although he had no pain during daily life activities.

The mean Lysholm knee score was 94.8 ± 6.8 (range, 78 to 100) at the latest follow-up. The score was more than 90 in 18 (82%) patients, 80 to 90 in 3 (14%) patients, and 78 in 1 (5%) patient who showed instability of the knee in physical examination and KT-1000 arthrometer measurement. The mean Lysholm score was 96.8 ± 4.5 (range, 84 to 100) in 16 patients whose interval from trauma to surgery was less than 30 days, and 89.3 ± 9.8 (range, 78 to 100) in 6 patients whose interval from trauma to surgery was more than 30 days (*p* = 0.134). The correlation between the degree of laxity measured by KT-1000 or Lachman test and the Lysholm scores was not statistically significant (*p* = 0.088 and *p* = 0.910, respectively*)*.

### Radiological outcomes

**Fig. 3:**
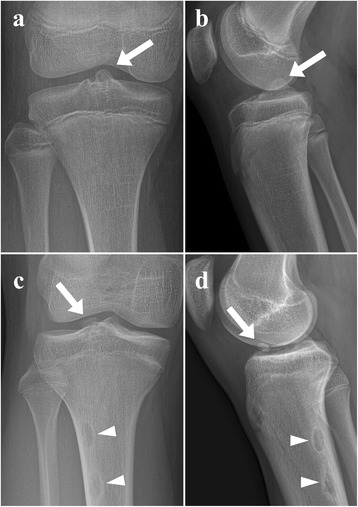
**a, b** Radiographs showing ledging (arrow), a prominent tibial eminence, possibly caused by malunion and overgrowth of the avulsed fragment. **c, d** In two patients, ossicles (arrow) were found above the tibial plateau. One patient had well demarcated intracortical osteolytic lesions (arrowhead) in the posterolateral aspect of proximal tibia

There was no case showing violation of proximal tibial physis by screws on postoperative radiographs. In 7 (26%) patients, radiologic abnormalities were found at the latest follow-up. Five (19%) had ledging which was prominent tibial eminence (Fig. 3). This might be caused by malunion and overgrowth of the avulsed fragment. Two (7%) were small ossicles above the tibial plateau (Fig. 3). Four of these 7 patients underwent operation at more than 30 days after the trauma. Extension deficit was not observed in these patients except one who underwent revision surgery due to re-displacement of avulsed fragment.

The length of the affected lower limb was 6.2 ± 5.9 mm longer (range, − 4.0 to 18.3 mm) than that of the contralateral lower limb measured against iliac crest height. It was 4.4 ± 4.7 mm longer (range, − 3.0 to 16.4 mm) measured against femoral head height. In 10 (50%) patients, the affected leg was more than 5 mm longer than the contralateral leg measured against iliac crest height. In another 10 (50%) patients, the difference in length between both legs was less than 5 mm. No patient whose contralateral leg was more than 5 mm longer than the affected leg. The mean length of femora of the affected side was 3.6 ± 4.1 mm longer (range, − 3.0 to 10.8 mm) than that of the contralateral side. The mean length of tibiae of affected side was 2.3 ± 3.4 mm longer (range, − 6.0 to 9.3 mm) than that of the contralateral side.

Associated knee injuries based on preoperative MRI and arthroscopic findings are summarized in Table 2. Six patients had lateral meniscal tear which was treated with partial meniscectomy (4 patients) or meniscal repair (2 patients). One medial meniscal tear was treated with partial meniscectomy.

**Table 2:** Details of the associated knee injury

*N* = 26							No.
None							14 (54%)
Bone contusion in the femoral lateral condyle							5 (19%)
Lateral meniscal tear							4 (15%)
Lateral meniscal tear and bone contusion in the femoral lateral condyle							1 (4%)
Lateral and medial meniscal tear							1 (4%)
Segond fracture							1 (4%)

## Discussion

The results show that arthroscopically assisted cannulated screw fixation is an effective and safe method for tibial eminence fracture in pediatric patients. Previous studies have described that fixation with a relatively large screw, compared to the small avulsed fragment, is technically demanding and can cause comminution of the bony fragment [28, 37, 38]. However, this complication was not found in this study. Union was achieved in almost all patients using a washer combined with a screw depending on the size of avulsed fragment and fracture pattern, consistent with prior results [32]. Other authors expressed concern that synovial leakage might occur after using a larger portal for a washer [27]. However, this complication did not occur in our series. To prevent posterior displacement of the avulsed fragment during screw insertion and consequent increased laxity, operators need to pay attention to the sagittal angle of the screw. Inserting two guide pins before screw insertion may help to prevent displacement and rotation of the fragment.

Arthrofibrosis developed in only one patient. This frequency is much less than the result of a previous multi-center study reporting an arthrofibrosis frequency of 10% in 205 patients treated with various surgical methods [14]. The authors immobilized the knee in full extension for 4 to 6 weeks in the majority of patients, which was longer than the 3 to 4 weeks in the present study. A previous systematic review reported that no patient developed arthrofibrosis in the screw fixation group compared to 6.3% of patients treated by suture fixation [39]. The data indicate that early range of motion exercise following firm fixation with cannulated screws might reduce the occurrence of arthrofibrosis.

A previous systematic review reported that 82% patients had laxity on both anterior drawer and Lachman test, while another systematic review reported abnormality on anterior drawer or Lachman test in 20% of patients [39, 40]. These rates are higher than presently observed. The discrepancy in instability might be attributable to a real difference of laxity or by heterogeneous criteria used to evaluate instability. In this study, side-to-side differences on physical examinations and KT-1000 arthrometer measurement were used to determine instability for pediatric patients, who are generally suppler than adults. However, another study used the absolute value on Lachman test to judge instability [33]. Similar to the results of other studies, there was no significant relationship between laxity and subjective symptom in the current study [33, 41]. However, a patient who had 5 to 10 mm of side-to-side difference on physical examinations and KT-1000 arthrometer also had pain and swelling of the knee after strenuous exercise and recorded the lowest Lysholm score.

High Lysholm knee scores have been reported, regardless of surgical methods or fixation devices [27, 39, 42, 43]. The scores were also high in our study. Since these uniform high scores might be due to a ceiling effect of Lysholm knee score [44], caution is needed when interpreting Lysholm score as a surgical outcome. In the current study, Lysholm scores of patients who underwent surgery more than 30 days after trauma were less than those of patients who underwent surgery within 30 days after trauma. However, their differences were not statistically significant. This might be due to type-II error.

Impingement of screw head on femoral condyle or trochlea has been rarely reported. A previous study of epiphyseal or transphyseal cannulated screw fixation related no case of chondral damage from a protruded screw [45]. Another case report introduced a patient who had 15° of extension deficit and chondral damage after an epiphyseal screw fixation with a toothed washer for type II fracture [46]. The authors assumed that use of a washer might have induced bulging of the screw head, resulting in extension impingement. However, a washer was not used in the two patients who had impingement in the present study. They underwent surgery at more than 30 days after trauma and two 5.0 mm screws were used. When the period between trauma and surgery is long or diameter of a screw is large, careful inspection for impingement should be made for the whole range of motion. Intercondylar notchplasty may be considered.

Nonunion rarely occurs after a tibial eminence fracture [39, 47]. Presently, only one patient underwent additional surgery due to nonunion. On the latest radiographs, 5 (19%) patients had prominent tibial eminence, consistent with results of a previous study showing 15% patients with ledging [27]. In the latter series, one of four ledgings caused an impingement phenomenon. In the current study, however, patients with ledging did not show extension deficit except one who received revision surgery.

Some previous studies have reported physeal growth disturbance after treatment for tibial eminence fracture [32–34, 39, 47, 48]. However, LLD was rarely evaluated. Ahn and colleagues described that 2 of 5 children have LLD of 1 cm (the affected legs is longer) after arthroscopic reduction and suture fixation [48]. In our study, the mean length of the affected leg was longer than that of the contralateral leg. Interestingly, both tibiae and femora of the affected side were longer than those of the contralateral side. Because LLD was not measured preoperatively, we cannot conclusively say that tibial eminence fracture is prone to occur in a longer leg or can make the affected leg longer.

Entrapment of the meniscus or intermeniscal ligament between the avulsed fragment and host bone can obstruct the reduction of a fracture [49, 50]. A study from the United States reported this finding after tibial eminence fracture in over 50% of patients [49]. Another Western study described that 59% patients have interposition of intermeniscal ligament under the avulsed fragment [30]. In our study of Korean patients, only 19% of the patients had these findings, although distribution of fracture type was similar to that in previous studies. In contrast, associated injuries including meniscal tear were more common in our study than those in Western studies [27, 33, 49]. A Japanese study also reported associated meniscal tear in 30% of patients [51], similar to our result. More data are needed to confirm this difference between Asian and Western patients.

This study had several limitations. First, because the study design was a retrospective case series, there was no control group treated with other methods. Second, results of some examinations, such as KT-1000 arthrometer and whole leg radiograph, in some patients could not be obtained due to their circumstances. This might have caused selection bias. Third, because the number of patients with type IV fracture was small, it was difficult to assess the outcome of cannulated screw fixation for type IV fracture. Fourth, Lachman and anterior drawer tests were performed manually without taking special landmarks, which may lead to low accuracy and reliability of these tests. And the result of pivot shift test which is also commonly performed in the patients with ACL insufficiency was not presented. Fifth, although we showed Lysholm score as a functional outcome scale, it would be better if we obtained Tegner or Marx activity scale together. Lastly, the sample size was not large, in some analyses, to verify the statistical significance.

## Conclusions

Arthroscopically assisted cannulated screw fixation for tibial eminence fracture in children and adolescents shows satisfactory clinical and radiological outcomes. Union can be achieved in almost every patient, and postoperative function of the knee is excellent. The occurrences of arthrofibrosis, instability, and screw head impingement are low, and the risk of violation of proximal tibial physis by screws are minimal.

## Abbreviations

ACL: Anterior cruciate ligament
AP: Anteroposterior
FC: Flexion contracture
FF: Further flexion
FHH: Femoral head height
ICH: Iliac crest height
LLD: Leg length discrepancy
S-to-S diff: Sde-to-side difference
RTA: road traffic accident
